# Late onset of the cryopyrin-associated periodic syndrome (CAPS) associated with low level of somatic mosaicism in six patients

**DOI:** 10.1186/1546-0096-13-S1-P37

**Published:** 2015-09-28

**Authors:** D Rowczenio, S Melo Gomes, J Aróstegui, E Omoyinmi, E Gonzalez-Roca, A Standing, D Eleftheriou, N Klein, P Brogan, H Lachmann, P Hawkins

**Affiliations:** 1 National Amyloidosis Centre, UCL Medical School, London, UK; 2 Institute of Child Health, UCL, London, UK; 3 Hospital Clinic-IDIBAPS, Barcelona, Spain

## Introduction

CAPS is caused by mutations in the *NLRP3* gene and is inherited in an autosomal dominant fashion. About 40% of children with CINCA are mutation negative by conventional Sanger sequencing, but *NLRP3* somatic mosaicism can be identified by sensitive multi-parallel sequencing (MPS) in a significant proportion of such patients.

## Objectives

To analyse the *NLRP3* gene in six patients with typical CAPS other than onset in mid-late adult life. All patients responded to IL-1 blockade and none had a family history.

## Methods

DNA was extracted from whole blood, saliva, buccal epithelial cells and from isolated monocytes, T and B lymphocytes and neutrophils. *NLRP3* gene was analysed using Sanger sequencing and MPS.

## Results

MPS detected a variable degree of somatic *NLRP3* mosaicism in all patients: two carried previously described variants p.E567K and p.A352T in 5.4% and 14.6% of alleles respectively; four had novel mutations: p.G569V, p.G564D and p.Y563C (found in two unrelated patients) in 21.1%, 5%, 5.1% and 11.1% of alleles respectively. Analysis of purified B and T lymphocytes, neutrophils and monocytes revealed a greater proportion of mutant cells among myeloid lineage; only a small fraction of T lymphocytes and buccal cells carried the *NLRP3* mutation. In a single adult patient who was heterozygous for germline *NLRP3* substitutions p.A439V and p.S434S, the mutation was present in all lymphoid and myeloid cells. We re-analysed the *NLRP3* gene in one subject who had been healthy until age 45, but had had relentlessly worsening CAPS and steadily increasing IL-1 inhibitor requirement, using a fresh sample obtained nine years after her initial assessment; this demonstrated an increase in the frequency of the mutant allele from 5.4% to 28.6% in DNA isolated from whole blood.

## Conclusion

These studies identified post-zygotic mutational events as the aetiology of late onset CAPS. All patients had excellent response to IL-1 blockade, including stabilisation of the amyloid load in the two subjects diagnosed with AA amyloidosis. AA amyloidosis is a severe complication of CAPS and hitherto has only been reported in patients with germline *NLRP3* mutations. Interestingly, the population of *NLRP3* mutant granulocytes and monocytes increased substantially in the single patient in whom a time course study was possible, by definition representing clonal expansion. Whilst further studies at the bone marrow level are planned, the current findings suggest that acquired *NLRP3* mutations may confer affected cells with a selective advantage.

